# WhatsApp in Stroke Systems: Current Use and Regulatory Concerns

**DOI:** 10.3389/fneur.2018.00388

**Published:** 2018-05-31

**Authors:** Juan M. Calleja-Castillo, Gina Gonzalez-Calderon

**Affiliations:** ^1^Neurology, Instituto Nacional de Neurología y Neurocirugía, México City, Mexico; ^2^Medicine, Instituto Tecnológico y de Estudios Superiores de Monterrey, Monterrey, Mexico

**Keywords:** teleneurology, stroke, smartphone, stroke systems of care, WhatsApp chat groups

## Abstract

Smartphone use is extremely common. Applications such as WhatsApp have billions of users and physicians are no exception. Stroke Medicine is a field where instant communication among fairly large groups is essential. In developing countries, economic limitations preclude the possibility of acquiring proper communication platforms. Thus, WhatsApp has been used as an organizational tool, for sharing clinical data, and for real time guidance of clinical care decisions. It has evolved into a cheap, accessible tool for telemedicine. Nevertheless, regulatory and privacy issues must be addressed. Some countries have implemented legislation to address this issue, while others lag behind. In this article, we present an overview on the different roles WhatsApp has acquired as a clinical tool in stroke systems and the potential privacy concerns of its use.

## Introduction

Smartphones are a hallmark of twenty-first century lifestyle. It is projected that 2.53 billion people will be smartphone users in 2018 (1). Numerous applications (apps) which allow immediate easy communication between physicians and health practitioners have been developed. They range from apps for instant messaging among the general population to full platforms that are specifically designed for clinical interaction among peers.

WhatsApp is an instant messaging application available for most smartphone operating systems. It was founded in 2009 and has become ubiquitous. It allows for free unlimited text messages, voice notes, voice calls, video conferences, and file exchange between users. It only needs an internet connection (WiFi or cellular network) and a smartphone to establish live communication with a person or group of people. It does not require a dedicated institutional network. Originally, the app was intended as a basic messaging app, but its versatility and cost has allowed it to become an important tool for co-worker interactions in health systems.

In developing countries with limited resources and overwhelmed healthcare systems, costly IT tools are beyond reach. Thus, inexpensive tools that allow more dynamic and efficient organization of the care team are greatly needed. Although these technologies can be implemented in several areas of clinical care, stroke systems benefit the most as it involves a multidisciplinary team and is faced with complex, time sensitive decisions.

In these countries, WhatsApp has spontaneously fulfilled the role as a clinical communication tool. Nevertheless, a concern regarding its use in medical care is the inadequate protection of private information.

The purpose of this perspective is to briefly review examples of currently available Instant Messaging (IM) applications, legislation about the use of these apps for clinical purposes in both developed and developing countries, to analyze the difficulties of implementing dedicated Electronic Health Records (EHR) IM applications in low-income settings, and to describe their current (and mostly unregulated) use in stroke systems of care.

## Law compliance and healthcare communication platforms

New apps known as smartphone-based paging applications have mostly replaced traditional paging systems when it comes to organization and communication among medical staff. The minimum desirable characteristic for these apps is to allow exchange of text messages that alert the clinician, assuring that issues such as battery life, network failure, or silent mode settings won't result in missed communications nor interfere with patient care. A clear advantage for smartphones is their ability to provide a more standardized record of these interactions, potentially incorporating them into the EHR and making them readily available for all members of the care team (2).

Mobile healthcare (m-health) applications with these characteristics are now being developed around the world. They aim to modernize the way in which medical staff communicates (3). Technologically speaking, it is not difficult to create a platform with the qualifications mentioned above. Shielding the very sensitive data frequently exchanged in these conversations is not as easy.

Several EHR software providers now include mobile applications to keep up with open IM applications. Their main advantage is that hospitals already using these platforms are more likely to keep doing so instead of switching to a new system that requires new staff training. Some of the most popular are Epic, InSync, Prime Suite, iPatientCare, ChARM EHR. All of them need to be acquired by the institution and have a monthly or yearly fee, and some charge per individual user. Some of these platforms are widely available, nevertheless, their expensive license fees cannot be afforded by health care systems in developing countries.

Another example is *Doximity*. It is the largest HIPAA (Health Insurance Portability and Accountability Act) compliant medical network in the United States, with more than 70% of U.S. doctors and thousands of nurse practitioners, physician assistants, and pharmacists as users. It enables instant connection with other healthcare professionals to securely collaborate on patient treatment^1^. Its resemblance to a social network also allows its users to grow their practices and engage on new career opportunities.

In the UK, an example is medCrowd, an app developed by medDigital, a group of local scientists and programmers. It provides a safe (National Health Service—England compliant) alternative for communication and sharing of clinical experience among teams and physicians in a local network or around the world^2^. It is an effort to globalize clinical discussion and to enable remote geographical sites with scarce resources to get involved and request advice from their peers about a clinical scenario. Similar apps used in the UK are Medicbleep, Hospify, and Forward.

Nevertheless, an app's impact depends on its popularity among the members of a community. Although some of these applications are easily reachable, their use is not widespread in countries other than the ones they were developed in, making WhatsApp an already established and more attractive option.

## Regulatory concerns and privacy issues for IM use in the healthcare setting

Personal information, especially that one related to healthcare is usually the object of extremely careful protection. All medical records contain sensitive data that could result in discriminative transgressions if made public. For this reason, laws have been established around the world to preserve the patients' right to confidentiality.

In the USA, there's specific legislation regarding patient confidentiality issues, the HIPA Act. Contrary to common knowledge, no communication platform is a 100% compliant with its standards, for safety of the information does not depend on the characteristics of the software but rather in how it is handled by its users.

WhatsApp has received plenty of attention recently because of its growing popularity among clinicians and healthcare practitioners. It is known to be efficient, and its use is certainly widespread, but the real question is: are we putting patients' information at risk? The unanimous answer from authorities on this matter is YES.

Before recent security measures were undertaken, exchange of clinical information through WhatsApp violated several national and international Copyright Laws (4). End-to-end encryption (E2EE) was introduced as a solution to this problem in 2014. When a text message is sent, it may pass through multiple servers before it reaches the intended recipient. E2EE turns the original message to coded chiphertext that can only be de-encrypted by a private key on the recipient phone, therefore no information can be retrieved from the intermediary servers (5).

Concerns exist now on whether the safety of end-to-end encryption's efficiency of WhatsApp data on Facebook servers is enough to make the platform HIPAA compliant. There are different forms in which information could leak from the app and thus, experts are not willing to consider it as safe as platforms designed for this purpose.

WhatsApp does not require an username or password to access data, other than the one on the device. Once the smartphone is unlocked, the information is vulnerable if it is left unattended. All messages and data are usually stored within the phone; when a person switches phones, the account will be preserved, but the information within the conversations needs to be backed up to be available on the new device. WhatsApp does not keep a record of delivered or deleted messages and there is no mechanism by which messaging interactions can be directly recorded in or accessed through the EHR, making it difficult for this information to be audited or peer reviewed.

Another issue may arise when the user leaves an employer (Hospital, Healthcare System, Clinic). The logistics of selectively retrieving and deleting clinical data would be complex. The only way to ensure that all electronic patient healthcare information is deleted from WhatsApp, is to eliminate the entire account. Since this app is also used for personal purposes, users would oppose to this alternative.

As a matter of fact, investigations about the employment of social networks on clinical medicine have been carried out by the General Medical Council, an independent organization whose aim is to protect patients and improve medical practice in the UK (6). Recommendations have been issued and, although most cases involved use of Facebook and Twitter by physicians, 3 cases did involve WhatsApp. Official advice from NHS England is very clear: “Whatever the other merits of WhatsApp, it should never be used for the sending of information in the professional healthcare environment^3^.”

As an example in developing countries, in Mexico, the regulatory authorities for the subject are the Ministry of Health (Secretaría de Salubridad y Asistencia) and the National Institute of Transparence, Information Access and Personal Data Protection (INAI) that issue the General Health Law^4^ and the Federal Law for Personal Data Protection (LFTAIPG)^5^, respectively. The General Board of Health Information (Dirección General de Información en Salud) published the NOM-024-SSA3-2012^6^ about legislation on electronic interaction between healthcare personnel. It establishes that all electronic communications must be established through an Electronic Health Record Information System (SIRES) that meets LFTAIPG requirements. This law is, nevertheless, seldom enforced.

Physicians and medical staff are frequently advised against the use of personal devices for clinical purposes by their institutions' ethics committees; however, all the regulations available apply to properly identified patient information (National Identification Number, name, date of birth) thus leaving a legal loophole for anonymized data.

The bottom line is that, in developing countries, WhatsApp use for clinical purposes is generalized, for the uses that have been detailed above, and stroke patients are not an exception. Economic, organizational, and social limitations make it difficult to enforce laws that may be ambiguous, and practicality usually prevails.

## Whatsapp as an organizational tool in stroke systems of care

In the USA and Europe, dedicated smartphone applications are being used for communication between stroke teams, groups of healthcare providers, and care coordinators. In developing countries, there are no economic resources in healthcare to allocate to such specific communication tools. Also, Information Technologies function with the minimally sufficient resources, lacks failsafe and are prone to breakdown (7). A recent online survey found that the main obstacles against IT use in health systems are poor infrastructure and lack of access to modern IT due to its high cost (8). IT budget is limited and it is being used preferentially in the implementation of EHR. Thus, there is a need for a free, easy-to-use communication tool for organizational purposes in hospitals.

WhatsApp is used in different stages of stroke patient care: In hospitals where pre-notification is not common, patients, and family members contact ER physicians and other specialists to alert them to their symptoms. Emergency medical services alert of a stroke patient they are currently transporting and report relevant information such as the result of clinical scales, time of onset of symptoms, and pertinent medical history.

At the hospital, WhatsApp is being used for direct communication between stroke program coordinators, physicians, and nurses for notification of the arrival and location of stroke patients throughout the hospital. When the stroke code is activated, patient information is immediately registered on the WhatsApp “Ictus Group.” The convenience of being able to inform several team members simultaneously proves to be advantageous. Included in this group are:

- Emergency medicine physicians and nurses in charge of first contact with the patient.
- Neurologists, who may not be physically present in the ER or even at the hospital but initiate transport as soon as they receive notice of the patient's arrival.
- Stroke Team Coordinators, who register the patient on the Stroke Program Database and will perform follow-up of the patient throughout their stay, immediately arriving at the ER and recording relevant information such as Door-to-Needle, Door-to-Groin times, and other quality performance measures.
- The radiologist and radiology technicians, who prepare the CT and MRI scanners for the patients' arrival.
- Rehabilitation specialists, who perform a physical therapy consult at a later date.
- Heads of department, who oversee the process as a whole.

Advantages of the use of a messaging service, such as WhatsApp, include the immediate and generalized awareness that a stroke patient has arrived, initiation of preparedness to receive the patient, knowledge for the whole group of the stage of evaluation/treatment the patient is in, and availability of patient data to the entire stroke team.

Disadvantages are mainly concerned with patient privacy and systematic attainment of patient data, as the non-standardized format of WhatsApp chats is amenable to different information being provided depending on who reports it.

## Whatsapp as a clinical discussion tool

The use of mobile devices for discussion of clinical cases is widespread in developing countries. Mobile health (m-health) offers a cheap alternative for teaching, clinical advise, and networking among peers (9). Nevertheless, proper information about its usage or evidence about its efficacy is virtually non-existent (10). The only randomized control trial that compared Emergency Department logistic outcomes with telephone vs. instant messaging app consultations showed a decrease in length of stay and in-person consultations in the group that used WhatsApp as a communication tool (11). Specifically, WhatsApp has become an universal tool for informal (and formal) discussion of cases among physicians in developing countries.

In Mexican Stroke Centers, its use is widespread. It is very common for clinicians to narrate a case to colleagues through a chat group. Usually these groups include:

- Physicians who practice a certain specialty, for discussion of interesting cases, where they usually include a brief roundup of the patient's history and examination, photos of relevant neuroimages and sometimes photographs or videos of the most important clinical phenomena.
- Groups of physicians of different specialties caring for a particular patient, where the group serves as a tool for updating the patient's clinical status, findings on examination, lab results and radiologic studies, and discussion of decisions regarding further treatment of the patient.

Among hospitals, it is not unusual to perform a sort of “tele-stroke medicine” in which an experienced stroke neurologist will provide guidance for decisions regarding thrombolysis to less experienced physicians. Patient's history, time of onset, clinical findings, NIHSS score, and CT scan images are sent via WhatsApp by the ER doctor, internal medicine specialist, resident or clinical fellow, and the stroke neurologist may advice on treatment alternatives.

## Conclusion

Technology trends advance rapidly, and rules and regulations may easily fall behind the versatility and ease of use of new resources. For stroke systems, a de facto use for WhatsApp is common, especially in developing countries. This platform may be very helpful in logistical organization, sharing of information, and “telemedicine,” specifically among stroke teams where attention is complex and time-sensitive. However, its use cannot go unchecked. A balance must be struck between cost, practicality, and protection of private information.

Personal mobile messaging has the advantage of being a low-cost, universally available application that can now share text, images, documents and audiovisual information. In stroke systems of care, it can help optimize organization and communication between its members. Although empiric coding of patient identifiers such as pseudonyms are often used by healthcare practitioners, guidelines for proper anonymization are missing. If WhatsApp is to be accepted as a safe and efficient clinical communication tool for stroke treatment, all these issues must be addressed.

## Author contributions

JC-C and GG-C were responsible equally of drafting the work and final approval of the version to be published and agree to be accountable for all aspects of the work in ensuring that questions related to the accuracy or integrity of any part of the work are appropriately investigated and resolved.

### Conflict of interest statement

The authors declare that the research was conducted in the absence of any commercial or financial relationships that could be construed as a potential conflict of interest.

## Footnotes

^1^Doximity - The Medical Network (2018) [updated 2018; cited 2018 January 13].

^2^medCrowd - Working Together (2017) [updated January 2018; cited 2018 January 13].

^3^NHS Information Governance Bulletin, (2015).

^4^Ley General de Salud, (2005).

^5^LEY FEDERAL DE TRANSPARENCIA Y ACCESO A LA INFORMACIÓN PÚBLICA, (2016).

^6^SISTEMAS DE INFORMACION DE REGISTRO ELECTRONICO PARA LA SALUD. INTERCAMBIO DE INFORMACION EN SALUD, (2012).
